# Protein-Water and Water-Water Long-Time Relaxations in Protein Hydration Water upon Cooling—A Close Look through Density Correlation Functions

**DOI:** 10.3390/molecules25194570

**Published:** 2020-10-07

**Authors:** Lorenzo Tenuzzo, Gaia Camisasca, Paola Gallo

**Affiliations:** Dipartimento di Matematica e Fisica, Università degli Studi Roma Tre, Via della Vasca Navale 84, 00146 Rome, Italy; lorenzo.tenuzzo@uniroma3.it (L.T.); gaia.camisasca@uniroma3.it (G.C.)

**Keywords:** hydration water, supercooled water, slow dynamics, molecular dynamics simulations, structural relaxations, hopping, glassy dynamics

## Abstract

We report results on the translational dynamics of the hydration water of the lysozyme protein upon cooling obtained by means of molecular dynamics simulations. The self van Hove functions and the mean square displacements of hydration water show two different temperature activated relaxation mechanisms, determining two dynamic regimes where transient trapping of the molecules is followed by hopping phenomena to allow to the structural relaxations. The two caging and hopping regimes are different in their nature. The low-temperature hopping regime has a time scale of tenths of nanoseconds and a length scale on the order of 2–3 water shells. This is connected to the nearest-neighbours cage effect and restricted to the supercooling, it is absent at high temperature and it is the mechanism to escape from the cage also present in bulk water. The second hopping regime is active at high temperatures, on the nanoseconds time scale and over distances of nanometers. This regime is connected to water displacements driven by the protein motion and it is observed very clearly at high temperatures and for temperatures higher than the protein dynamical transition. Below this temperature, the suppression of protein fluctuations largely increases the time-scale of the protein-related hopping phenomena at least over 100 ns. These protein-related hopping phenomena permit the detection of translational motions of hydration water molecules longly persistent in the hydration shell of the protein.

## 1. Introduction

The water around a biomolecule is called hydration water and for proteins this water is particularly important for their correct functioning [1]. Without a certain level of hydration water a protein can not perform its biological functions. Moreover, hydration water stabilizes the protein structure and mediates interactions between biomolecules. For these reasons it has been subject of many studies, see for example References [2,3].

In the last years, evidence of the fact that hydration water exhibits two structural relaxations has grown. Experimental works performed at ambient temperature by means of extended depolarized light scattering techniques [4,5] on dilute water solutions of proteins and other biomolecules, have clearly distinguished two slow relaxations, whereas in pure water only one is present. Of these two relaxations, one exhibits the time scale of the typical α-relaxation, the structural relaxation that bulk water shows upon cooling [6,7,8], while the second one is slower. The second slower relaxation was assigned to the hydration water contained in the solution, also because its timescale actually depends on the complexity of the bio-molecule [5,9]. The same two relaxations were also detected by studying the density fluctuations of the water contained in different sugars solutions by means of molecular dynamics simulations [10]. Broadband dielectric spectroscopy measurements also showed two relaxations and highlighted the coupling of the slower one to the solute dynamics [11]. These experiments show indirect evidences as they average over the hydration water and the bulk-like water contained in the systems. Experiments probing restrictively the dynamics of the hydration water alone were performed by employing intrinsic fluorescent probes [12,13] or 2D infrared spectroscopy [14,15] and detected two relaxations belonging only to hydration water. The slower time-scale depends on the protein site, which is consistent with the broad residence time distributions at protein-interfaces found in other studies [16,17,18].

The knowledge about the second relaxation of hydration water in connection to the protein dynamics has greatly benefited by those studies of dilute biosystems performed upon cooling. When the dynamics of the hydration water is probed as a function of the temperature, especially down to the supercooled regime of water, new dynamical features are observed both in the protein and hydration water dynamics and from them it is possible to infer the coupling between the protein internal motion with its hydration water. In fact, proteins undergo a dynamical transition, called protein dynamical transition (PDT), a phenomenon in which proteins increase their flexibility suddenly with increasing temperature only if hydrated. The PDT takes place in the supercooled regime of water [19,20,21,22].

Quasi-elastic neutron scattering experiments and molecular dynamics simulations on lowly hydrated protein powders [23,24,25,26] detected the presence of a relaxation process showing a dynamic crossover in coincidence of the PDT.

Molecular dynamics simulations in combination with calculations of density-density correlation functions in the *Q*-space enabled to study selectively the translation dynamics of the protein hydration water alone [27,28], in range of high hydration levels, thus reproducing biological water-rich environments. These studies explicitly showed that the slow time decay of density correlators comes from two distinct relaxations. By comparing the time-scale and the temperature behaviour of these two relaxations, it was shown that the faster relaxation is the analogous of the α relaxation of bulk water, and consequently it was termed α relaxation of hydration water. The second slower relaxation, termed long relaxation of hydration water, exhibits a different temperature behaviour upon supercooling with respect to the α process and, most importantly, it was shown to be sensitive to the PDT. The loss of structural fluctuations of the protein below the PDT directly contributes to a change of behaviour as a function of temperature, of the long relaxation of the involved hydration water. This clear crossover between two different regimes happens exactly at the PDT of the studied protein.

Although the presence of two relaxations of protein hydration water is recognised in the literature, much still needs to be done particularly in order to characterise the second slow relaxation of hydration water. In this study, we analyse the single particle density-density time correlation function in real space, the Self van Hove Function (SVHF), which probes translational dynamics. These correlation functions are calculated for the hydration water of the lysozyme protein, which is immersed in water in dilute conditions. We study this system by means of classical full-atom molecular dynamics (MD) simulations. We characterised the translational dynamics of hydration water probed in the reciprocal space and upon cooling in a series of previous works [27,28,29] and at T=300 K in the real space in Reference [30]. Here we extend our study on protein hydration water in the direct space with new, longer, simulations of 100 ns for temperatures ranging from T=300 K down to the supercooled regime and until T=200 K.

The results on the two relaxations of lysozyme hydration water detected in the density-density time correlation functions in *Q* space and their temperature behaviour give us a working background for this new study and we briefly review them in Section 3. In Section 4 we present the new results on the SVHFs. After presenting the correlators, we focus the attention on the two slow relaxations and on their different mechanisms corresponding to the long time behaviour of the SVHFs. The bulk-like slow α relaxation is analysed for hydration water in Section 4.1 upon cooling. The long relaxation that is characteristic only of hydration water is analysed in Section 4.2. In Section 4.3 we discuss the mean square displacement of hydration water in order to independently determine the length and time scales of the two mechanisms that cause the slow dynamics of hydration water. In Section 5 we draw our conclusions.

## 2. Methods

**Figure 1:**
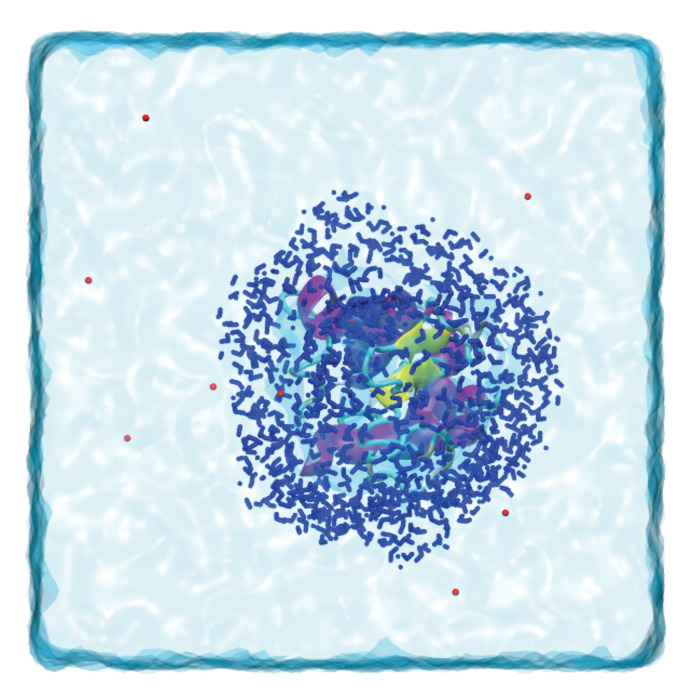
Snapshot of the system at T=280 K: hydration water is shown in licorice structure (balls + sticks) and colored in blue, protein is depicted and colored highlighting its secondary structure, the rest of the water in the box is shown as a transparent surface. Cl${}^{-}$ ions are shown in van der Waals representation and colored in red.

The system under investigation consists of one lysozyme protein immersed in liquid water shown in Figure 1. The total number of water molecules is 13,982 and 8 chlorine ions are added to the liquid bulk to counterpoise the positive charges of the residues of the protein so that the total charge of the system is zero.

Classical all-atoms MD simulations are run to investigate the dynamics of the water proximal to the protein. The system is studied at pressure p=1 bar for temperatures ranging from 300 K down to 200 K. The CHARMM force field was used to model the lysozyme and the ions and the SPC/E potential was used for water. The cut-off value for the non-bonded interactions was set to 10 Å and the electrostatics were handled with the particle-mesh Ewald method. Equilibrated configurations of the system at each temperature were taken from previous studies [27,28] and simulations were extended of a length of 100 ns for each temperature. Details of the preparation of the system and simulation protocols can be found in the cited literature. On these new 100 ns-long simulations, self van Hove functions and mean square displacements were calculated considering only the contribution of hydration water, defined as the group of water molecules moving within a shell of 6 Å from the protein.

## 3. Slow Dynamics of Hydration Water

The translational dynamics of the hydration water of lysozyme have been characterised upon cooling in a series of our previous studies [27,28,29,30]. We summarize here the main findings. The dynamics was probed by calculation of the Self Intermediate Scattering Function (SISF) which is the time auto-correlation function of the microscopic density in the *Q*-space. The SISF, $F_{SELF}\left(Q,t\right)$, is defined as:

(1) $$F_{SELF}\left(Q,t\right)=\frac{1}{N}\langle \sum_{i}e^{-i\vec{Q}\cdot \left[{\vec{r}}_{i}\left(t\right)-{\vec{r}}_{i}\left(0\right)\right]}\rangle ,$$

where *N* is the number of water molecules and ${\vec{r}}_{i}\left(t\right)$ the position of the oxygen atom of the *i*-th water molecule at time *t*. The brackets $\langle \cdots \rangle$ indicate the average over time origins. This function is particularly suitable to study the translational dynamics of liquids in the supercooled regime, as the different microscopic mechanisms characterising the dynamics can be tracked by analysing the shape of this correlator.

**Figure 2:**
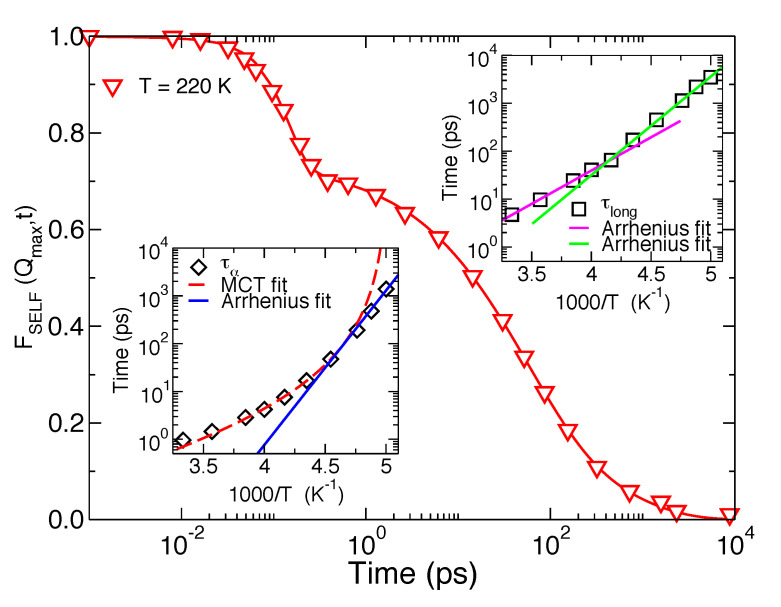
Main panel: $F_{SELF}\left(Q_{\max},t\right)$ of lysozyme hydration water oxygens calculated at T=220 K. The continuous line is the fit to Equation (2). Bottom inset: behaviour of the $\tau_{\alpha }$ as a function of the temperature. At high temperature it fits the Mode Coupling Theory (MCT) (fragile) behaviour (dashed line), while at low temperature it fits the Arrhenius (strong) behaviour (continuous line). The fragile to strong crossover is observed at T=215 K. Upper inset: behaviour of the $\tau_{long}$ as a function of the temperature. It fits to two Arrhenius laws with different activation energies at high and low temperature. The strong to strong crossover is observed at T=240 K where the lysozyme shows its protein dynamical transition (PDT) (not shown [28]).

The SISFs of hydration water have been calculated directly from the MD trajectories for the oxygen atoms of the water molecules moving inside the 6 Å-thick shell around the lysozyme protein for temperatures ranging from T=300 K down to T=200 K. In Figure 2 we report the SISF of hydration water at T=220 K showing the relevant phenomenology of the supercooled regime. This function was calculated at $Q=Q_{\max}=22.5$ nm${}^{-1}$, where the structure factor of water shows its principal maximum and the features of the slow dynamics in the supercooled regime are best detected [31].

We observe the first decay of the curve happening at short time, which corresponds to the ballistic regime: at such short times the tagged water molecule is still far enough from the neighbour molecules and in fact it moves as a free particle. As the time increases, the tagged water molecule starts feeling the interactions with the surrounding environment because it approaches spatially the neighbours. Due to the low temperature it remains trapped inside the cage the neighbours form around it. The motion of such trapped molecule corresponds to a rattling motion inside the cage and this situation corresponds to intermediate times, ∼1 ps in Figure 2, where the $F_{SELF}\left(Q_{\max},t\right)$ settles on a plateau value: here correlations persist in time. This intermediate cage-regime and the duration of the plateau region depend largely on the temperature, the cage regime is in fact absent at high temperature and extends over several orders of magnitude when the system approaches the glassy state [31].

In the liquid state the cage eventually relaxes and the tagged water molecule is free to explore other locations. In the case of pure water, this molecule will reach a diffusive regime while exploring the bulk region with the $F_{SELF}\left(Q,t\right)$ decaying to zero through a single process called α-relaxation [6,7]. The α-relaxation is the structural relaxation of liquids described in the mild supercooling region by the Mode Coupling Theory (MCT) [31] for many glass formers including water [6,7,8]. In the case of hydration water, after the cage-regime the hydration water molecule will explore other locations over the protein surface and will reach a sub-diffusive regime [30]. Correspondently the long-time decay to zero of the $F_{SELF}\left(Q,t\right)$ is due not only to the α-relaxation but also to a second long relaxation which arises from the dynamic coupling of the protein with its hydration layer [28]. The relevant time-scales of the α and long relaxations, $\tau_{\alpha }$ and $\tau_{long}$, can be extracted directly from the SISFs by fitting the curves to the model [10,27]:

(2) $$F_{SELF}\left(Q,t\right)=\left(1-f_{\alpha }-f_{long}\right)e^{-{\left(t/\tau_{s}\right)}^{2}}+f_{\alpha }\;e^{-{\left(t/\tau_{\alpha }\right)}^{\beta_{\alpha }}}+f_{long}\;e^{-{\left(t/\tau_{long}\right)}^{\beta_{long}}}.$$

In Equation (2), the Gaussian term takes into account the short-time ballistic regime, while the two stretched exponential functions model the α and the long relaxation respectively. The long term is essential to reproduce the long-time stretched tails of the correlators of hydration water which can not be described by the model used to describe the density correlators of pure water [6,7]. The latter comprises only the first two terms of Equation (2), being the amplitudes of these two terms normalized to 1.

Both structural relaxations of hydration water have been characterised in temperature. In the insets of Figure 2, the temperature behaviour of $\tau_{\alpha }$ (bottom) and $\tau_{long}$ (upper) are shown in two Arrhenius plots. Upon cooling, $\tau_{\alpha }$ can be described by a power law of the form $\tau_{\alpha }∼{\left(T-T_{C}\right)}^{-\gamma }$, with $T_{C}=199$ K and γ=2.68. This is the behaviour predicted for the α relaxation by the MCT and liquids whose structural relaxation times follow this temperature dependence are classified as fragile liquids. At low temperature, the temperature behaviour of $\tau_{\alpha }$ is instead well described by an Arrhenius law of the form $\tau_{\alpha }∼e^{E_{A}/\left(k_{B}T\right)}$, with $E_{A}=61.9$ kJ/mol. Liquids with structural relaxation times following the Arrhenius law are classified as strong liquids. $E_{A}$ is the activation energy that characterises activated (hopping) processes that start when cages are frozen and that are not taken into account by the ideal version of the MCT. Therefore, a dynamic crossover from a fragile to a strong regime occurs, approximately at T=215 K, in the α-relaxation of the hydration water of the lysozyme protein [28]. The α relaxation of pure water shows the same phenomenology and the same time scales, with the fragile to strong crossover happening at T=210 K for SPC/E bulk water [32]. In bulk water, the fragile to strong crossover is related to the crossing of the so-called Widom Line, the line of maxima of response functions emanated from a critical point [33]. The long relaxation, that is missing in bulk water, shows a different temperature behaviour with respect to the α process. It can be described by two Arrhenius laws, with different activation energies at high temperature (26.6 kJ/mol) and low temperature (39.3 kJ/mol). The crossover between these two strong regimes is termed strong to strong crossover and it happens at T=240 K. Moreover, this relaxation which happens over a longer timescale with respect to the α one, has been shown by our studies to be coupled with the protein internal dynamics. Specifically, the PDT of lysozyme can be detected in MD simulations by quantifying the protein structural fluctuations for the hydrated lysozyme. This can be done by plotting its MSD as a function of temperature and detecting the crossover between a steeper and a gentler slope. This happens at T=240 K, therefore in coincidence with the strong to strong crossover of $\tau_{long}$, as shown in Figures 6 and 7 of Reference [28].

## 4. Results

The Self van Hove Function (SVHF) is the single particle density-density correlation function in the real space. It is defined as:

(3) $$G_{SELF}\left(\vec{r},t\right)=\frac{1}{N}\langle \sum_{i}\delta \left(\vec{r}-\left[{\vec{r}}_{i}\left(t\right)-{\vec{r}}_{i}\left(0\right)\right]\right)\rangle .$$

In this study, we calculated the radial part of Equation (3), $4\pi G_{SELF}\left(r,t\right)$, which is the probability density of a displacement *r* in a time interval *t*. The detailed description of the average evolution of hydration water molecules in time and space it is possible by evaluating the radial part of Equation (3) by considering the positions of the oxygen atoms of the water molecules moving inside the hydration shell of lysozyme. Besides, the SVHF and the SISF are Fourier transform pairs, and therefore the study of the SVHF gives complementary informations about the relaxation mechanism of hydration water. SVHFs have been calculated for water simulated with different model potentials [7,34,35,36] and recently measured with X-ray scattering experiments [37].

Generally speaking, the SVHF is the distribution of the particle displacements that took place in an interval of time *t*. When the dynamics follows the MCT theory, the general shape of the correlator as a function of the time is unimodal with the single peak shifting toward large distance and spreading. When cages are frozen in many glass formers an atom or a molecule spatially trapped by its surrounding neighbours can escape by hopping on particular allowed, energetically favoured, distances. These phenomena are also called activated processes. When these processes dominate the relaxations, due to the heterogeneity of the dynamics of the particles, the SVHF can assume a multimodal shape, that is, it can develop multiple peaks and shoulders, meaning that different groups of molecules experience different dynamical regimes. The cage effect and the hopping phenomena due to the supercooling exist in many glass formers and have been observed through the SVHF, see for example References [38,39,40]. They have been observed also for bulk water [7,35], and in sugar-water solutions [41]. In Reference [30] we started to study of the SVHF of lysozyme hydration water at T=300 K, on MD trajectories of 20 ns. There we found for hydration water a new hopping regime, not connected to the glassy behaviour that develops upon supercooling, but due to the protein rearrangements instead.

Here we extend this study in the supercooled regime, with new longer simulations in order to better characterise the complexity of these slow relaxation phenomena of protein hydration water.

### 4.1. Van Hove Correlation Functions of Hydration Water for the Short Time Dynamics and the α Slow Dynamics

**Figure 3:**
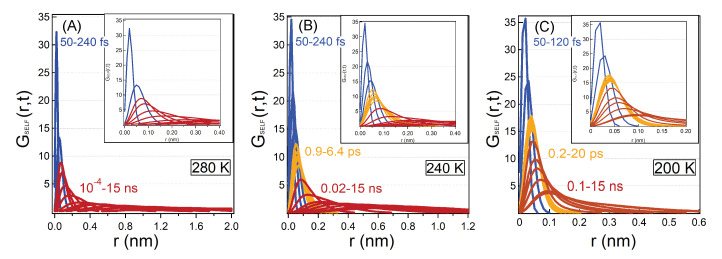
Self van Hove Functions (SVHFs) calculated for three temperatures: T=280 K (panel **A**), 240 K (panel **B**) and 200 K (panel **C**). The chosen curves evidence the water relaxations focusing in particular on the ballistic behaviour, the caging and the α relaxation of the MCT. Colours are used to point out three different time regimes which characterise the dynamics of the system. At short times (blue curves), the particle moves as a free particle with a ballistic behaviour and it is found for all the temperatures. At intermediate times and sufficiently low temperatures (orange curves), water is trapped in the transient cage, and this phenomenon is already present at T=240 K and becomes particularly evident at T=200 K where the transient cage lasts for a long time. In the long time regime (red curves) density fluctuations show the α relaxations process similar to bulk water. This relaxation process is well described by the MCT theory. In the insets of the figure we show a blow-up at intermediate times to best underline the MCT cage effect.

In this section, we present and discuss the SVHFs of hydration water calculated at different correlation times and temperatures. In the three panels of Figure 3 the SVHFs of hydration water are reported for three temperatures T=280 K, 240 K and 200 K. In each panel, correlation times cover six orders of magnitude spanning from t=56 fs to 15 ns.

At short time the motion of hydration water is led by the fast relaxation of the ballistic regime, the SVHFs are narrow and highly peaked at short distances and their maximum moves to larger distances ballistically up to 240 fs. This regime does not depend much on the temperature. The ballistic regime is followed by the structural relaxations that stretches in time as supercooling progresses and correspondingly the curves continue to shift to larger distances and spread. At very long time, the curves show a multiple peaks shape, we will discuss this in more detail in the Section 4.2.

At the two lowest temperatures shown in the picture, an intermediate regime of the dynamics appears: we observe that the curves, after the ballistic regime, group together for several picoseconds. This corresponds to the cage-effect induced by the supercooling, where the particle remains trapped inside the cage formed by its neighbours, correspondently the curves do not show temporal evolution as time progresses. The particles are trapped within a linear distance from the original position of ∼0.05 nm, which is well inside the position of the first peak of the radial distribution function. This estimate is also in accordance with the cage radius that can be extracted from the plateau of the SISFs of hydration water, $a∼-3\sqrt{\ln(\phi )}/Q_{max}^{2}=0.046$ nm, where ϕ∼0.7 is the value of the plateau in the SISFs. The same cage radius was also determined in bulk water [7].

Eventually the relaxation of the cages permits the hydration water molecules to proceed in their motion and the SVHFs start to evolve again towards large distances. All the curves in Figure 3, except the ones for long correlation times t>5 ns at 280 K, are unimodal distributions and follow the predictions of Mode Coupling Theory [31]. We will concentrate on the high temperature non unimodal distributions in the next section as they are not related to the bulk-like glassy behaviour that we analyse now in this section.

From the comparison among the panels of Figure 3, it is also possible to appreciate the slowing down of the motion of hydration water molecules upon supercooling: at a fixed correlation time the particle travels less upon decreasing the temperature. In fact both the α and the long relaxations are slowed down (Figure 2).

**Figure 4:**
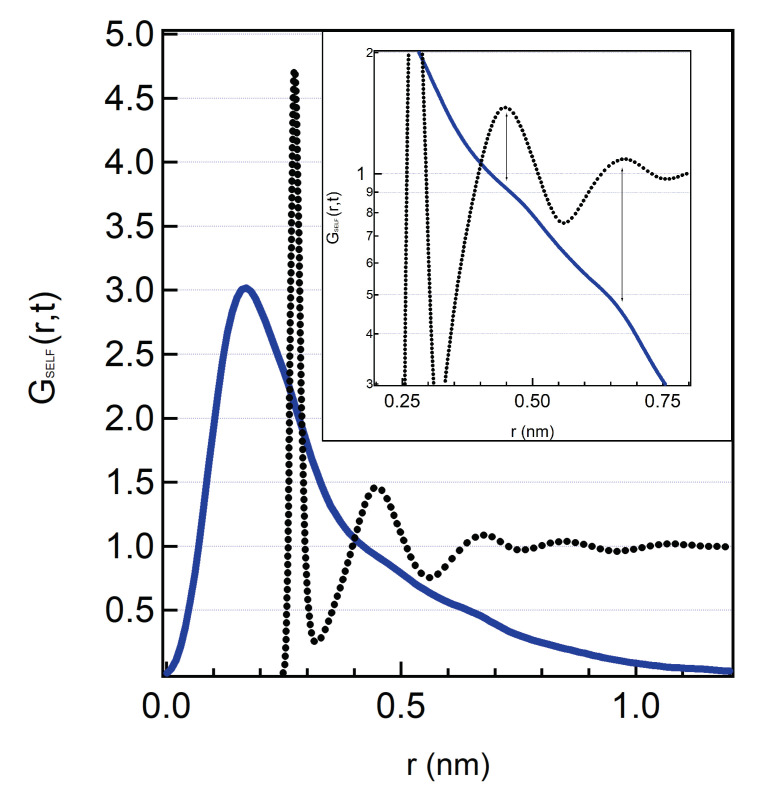
SVHF (blue continuous line) for hydration water at T=200 K for a long correlation time, t=50 ns. In this curve it is evident the presence of hopping phenomena: the shoulders that can be seen in the curve are due to the jumps of the particle out of the molecular cage. The dashed line represents the radial distribution function of the oxygens of bulk water: the shoulders correspond to the location in space of the second and third shells around the particle, as best evident in the inset, where we show a blow-up of the same curves in semi logarithmic scale.

We now focus on a temperature below the MCT-crossover temperature, T=200 K where cages are frozen and in bulk water hopping processes restore ergodicity. In Figure 4 we show the SVHF of hydration water at T=200 K calculated at a value of the correlation time extremely long, t=50 ns. In the same figure it is also reported the radial distribution function of bulk SPC/E water at the same temperature and pressure from which we can easily evaluate the positions of the first three shells around the central water molecule. In Figure 4 it is visible the hopping phenomenon that is not predicted by the ideal MCT theory but it is included in its extended version. In particular, upon approaching the MCT crossover temperature $T_{C}$ from above, thermal fluctuations become small enough to prevent the cage relaxation and the particle would remain trapped causing structural arrest of the liquid. Nonetheless, we do observe translational motion of the water molecules at low temperature: this is due to the activation, already few degrees above $T_{C}$, of the new relaxation mechanisms called hopping phenomena. Hopping phenomena permit the structural relaxation at low temperatures and their activation results in a strong regime for the α relaxation. To escape the cage, water molecules hop to energetically favoured positions which corresponds to the distances of the neighbours. This process is visible (see the inset of Figure 4) by the alignment of the shoulders of the SVHF with the peaks of the oxygens radial distribution function. The same hopping phenomena are also observed in bulk water [35].

### 4.2. Van Hove Correlation Functions of Hydration Water for the Long Time Dynamics

**Figure 5:**
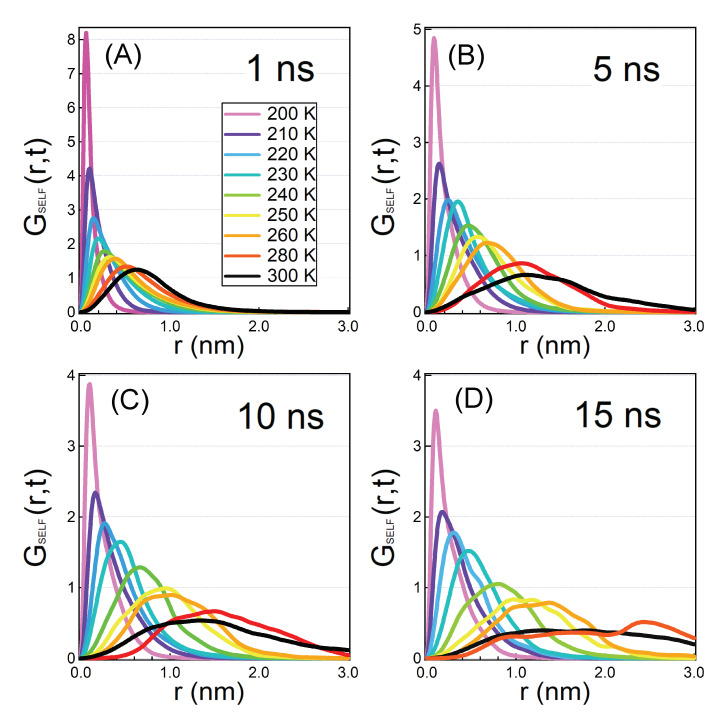
Long time SVHFs calculated from T=200 K to 300 K for oxygens of hydration water. The panels correspond to four different correlation times: t=1 ns (**A**), t=5 ns (**B**), t=10 ns (**C**) and t=15 ns (**D**). From 1 ns to 5 ns the curves mainly show the bulk-like MCT water dynamics. From 5 ns to 15 ns we start to see new hopping phenomena. At variance with the bulk-like hopping phenomena described in the previous figure, these hopping phenomena appear already for high temperatures, and the peaks are very well defined and present for lower and lower temperatures as time becomes longer.

In Figure 5 we show the temperature evolution of SVHF of hydration water at all the simulated temperatures at four long correlations times. By comparing the curves at fixed correlation time it is possible to quantify the slowing down of the motion of hydration water induced by the low temperature: every curve is lower and wider with respect to the curve calculated at lower temperature. Moreover, by comparing curves calculated at different correlation times and at a fixed temperature, we observe that at high temperatures the curves continue to evolve in time since their shape changes, while at low temperature the curves do not change much even though we are looking at very long correlation times. For example, at T=200 K the peak of SVHF is found at ∼0.2 nm both at t=5 and 15 ns.

At t=1 ns all the distributions show a smooth shape and are unimodal. At t=5 ns, in the curve corresponding to T=300 K two shoulders at larger distance with respect to the main maximum develop. This phenomenon becomes more marked and it extends to more curves if we look at the longer correlation times. In particular, shoulders affect clearly the curves from T=300 K down to the lowest temperatures at t=10 and 15 ns. Some of the curves, and more evidently at higher temperatures and longer correlation times, appear therefore to have lost the unimodal regular shape which characterises them at earlier times.

**Figure 6:**
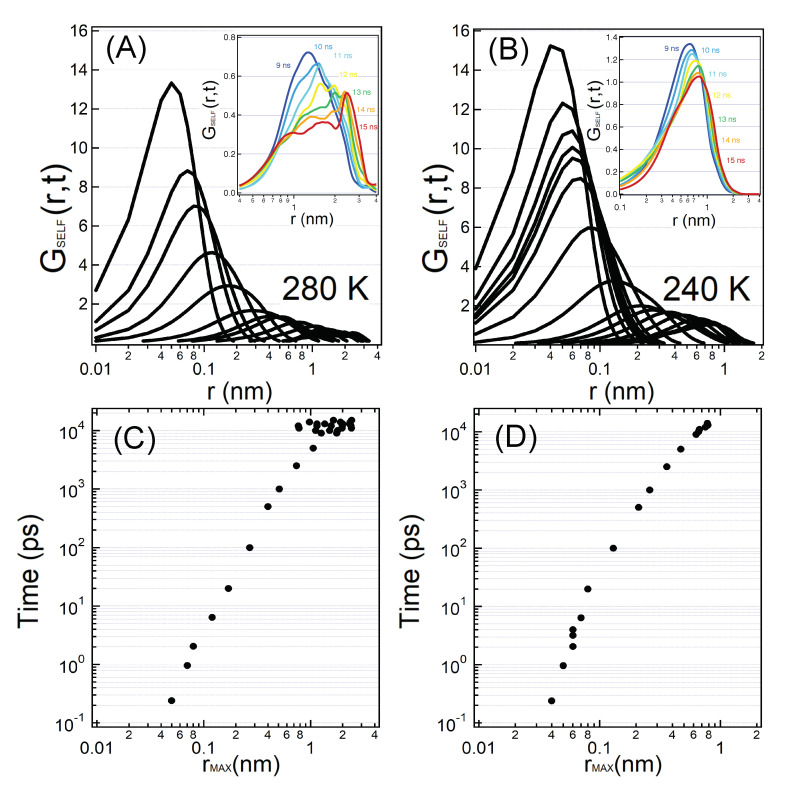
In order to best evidentiate the high temperature hopping phenomena, here there are plotted the hydration water oxygens SVHFs in logarithmic scale at T=280 K (panel **A**) and T=240 K (panel **B**) and the position of the peaks of the curves as a function of the time at T=280 K (panel **C**) and T=240 K (panel **D**). At T=280 K we clearly observe the presence of multiple peaks concentrated between 9 and 15 ns. This long relaxation due to the protein motion is much slower than the α relaxation at the corresponding temperature. At T=240 K we mainly observe the evolution of the α peak which moves over longer distance over time. For temperatures lower than T=250 K (at the PDT and below) we have more difficulties to observe the hopping that determines the strong behaviour of the long relaxation because the long relaxation shifts to times that are long compared to the timescale of our simulations.

To best point out the position of the shoulders we report in the upper panels of Figure 6 the SVHFs at T=280 K and T=240 K for correlation times ranging from t=240 fs to t=15 ns. In the bottom panels the correlation times at which we observe the main peak and its shoulder(s) are reported as a function of the positions of these features. At T=280 K we find a single peak of the SVHF of hydration water up to ∼9 ns, for later times multiple shoulders appear. Some curves, such as the one calculated at t=12 ns, have up to four different shoulders and therefore locations in space where the water molecule has a probability to have hopped. At times t=13, 14 and 15 ns the peak at 2.5 nm becomes sharper and sharper. The positions of these shoulders span from 0.8 nm and 3 nm. At T=240 K, we see a cage regime between 2 ps and 6 ps where the single peak of the SVHF of hydration water does not move in time, beyond this regime we observe a single main peak moving toward large distances. At variance with respect to T=280 K, the SVHF does not develop shoulders in the time range investigated in this figure.

**Figure 7:**
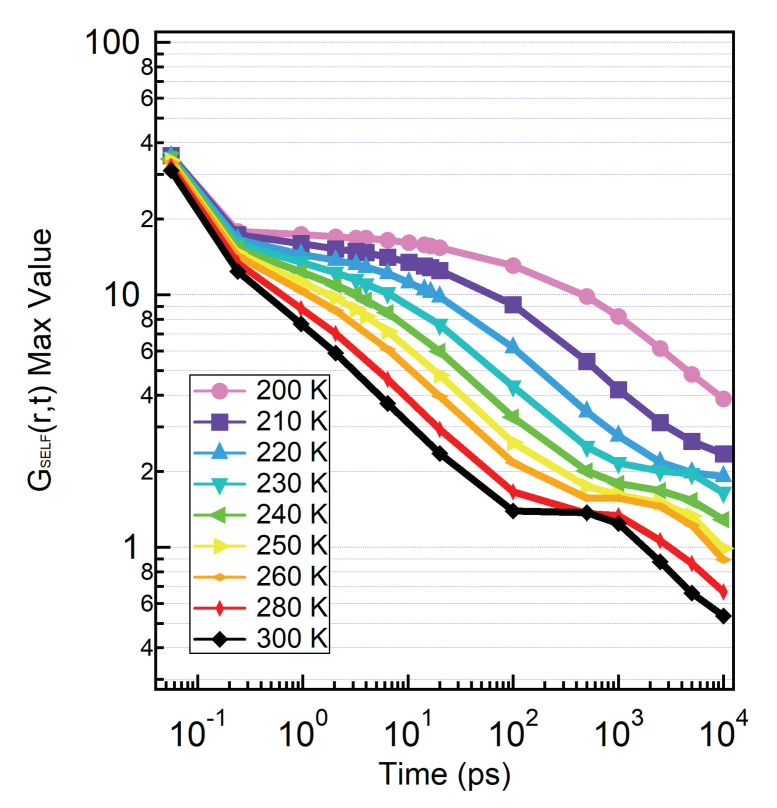
Value of the main maximum of every SVHFs over time. At low temperatures we can see the presence of a plateau, due to the *cage effect*. At the highest temperature the relaxation appears to follow the α relaxation until 100 ps. From 100 ps to 1 ns we can see a new plateau which shifts at longer times upon cooling. This effect is due to a *protein cage effect* and it is also related with the PDT.

We said in Section 4 that the multimodality in the SVHFs reveals dynamical heterogeneities in the displacements of particles and are called generally hopping phenomena. We also pointed out the characteristic features of the hopping mechanism which allows the water molecules to escape from the frozen cages (α relaxation in the strong regime) in Section 4.1. Nonetheless, the present hopping phenomena are different from the hopping phenomena of the α relaxation. We observe in fact that (i), the development of shoulders in the SVHF affects the large distances, being their positions at 0.8–3 nm, where the radial distribution functions approach unity, and thus distances well beyond the typical inter-particle distances of few water shells; (ii), the present multimodality of the SVHFs arises at high-temperature where there is no MCT cage-effect; (iii), the time scale at which this phenomenon appears (>ns) is much larger than the α relaxation regime at that temperature. The new hopping phenomena of Figure 6 and Figure 7, are connected to the long-time protein internal motion such as relative motions of globular domains, and to the long relaxation of hydration water molecules with very high residence time inside the shells.

We note also that we observe these high-temperature long-time hopping phenomena mainly at and above T=250 K, the PDT of our lysozyme protein. The protein movements are in fact strongly hindered below the PDT.

For temperatures lower than T=250 K ( below the PDT) we cannot follow well the hopping that determines the strong behaviour of the long relaxation because below the PDT protein motions are slower and the long relaxation times of hydration water related to the protein motions are too long, given the length of our simulations, to follow. We recall that at the PDT this long relaxation has a strong to strong transition and the relaxation times grow very much below the transition temperature. At low temperatures in the time region that we can investigate we showed that we are sensitive to the hopping due to the cage-effect that is a different hopping that activates only at low temperature.

### 4.3. Two Caging Regimes and Mean Square Displacement

To further investigate this long time mechanism that is present only for hydration water, in Figure 7 we plot the value of the main maximum of SVHFs of hydration water as a function of the correlation time. Each curve corresponds to a different temperature, from T=300 K down to T=200 K. The decay to zero of the main peak provides information complementary to the SISFs. We observe a short time fast-decay corresponding to the ballistic regime. At low temperatures the curves develop the typical plateau due to the glassy cage-effect, already evident in the SISFs. The plateau starts at ∼250 fs and extends up to ∼10 ps at the lowest temperature. These estimates for the cage-regime are in agreement with time intervals over which the SVHFs group together in Figure 3. After the ballistic regime at high temperatures, or after the cage-regime at lower temperatures, the hydration water molecules enters the α and then the long relaxation regime and the main maximum of the SVHFs decays toward zero.

We observe here a novel feature which is the development of a second plateau region, a feature that was not evidenced in the SISFs calculated at a fixed *Q*. We can appreciate from the figure that the maximum of the SVHFs is able to track the relevant time scales in time. The plateau is observable at T=300 K in the long interval from t=100 ps up to t=1 ns. As the temperature decreases, the second plateau develops later in time, and we can distinguish it clearly down to T∼230 K. Similar to the MCT caging also this behaviour precedes the escape of the water molecules from the *protein cage*, in this case detected through the development of shoulders in the SVHFs that define the related hopping region.

To further characterise the translational motions of hydration water molecules we also analysed the mean square displacement (MSD). The MSD is defined as:

(4) $$\mathrm{MSD}=\frac{1}{N}\langle \sum_{i}{\left|{\vec{r}}_{i}\left(t\right)-{\vec{r}}_{i}\left(0\right)\right|}^{2}\rangle .$$

**Figure 8:**
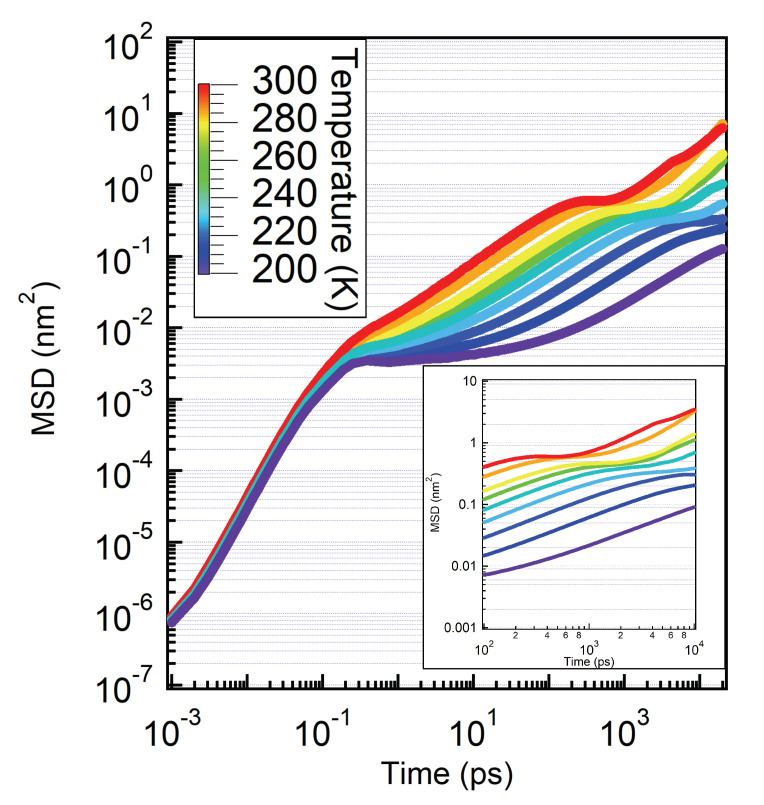
Mean Square Displacement. Particles shows a sub-diffusive behaviour at high temperatures until they reach a new plateau that starts at 100 ps for T=300 K. This feature is related to the plateau that we see in Figure 7 and which is different from the one that we observe for the low temperatures, which is related to the MCT caging.

We calculated the MSD of hydration water by considering the oxygen positions in Equation (4) and we show the results in Figure 8.

Also in these correlation functions we can distinguish the different dynamical regimes of hydration water: the ballistic regime, weakly dependent on the temperature, up to 250 fs where the MSD follow the predicted $\propto t^{2}$ behaviour. For the lowest temperatures, this is followed by the MCT cage regime: here we observe a plateau region between 1 ps up to 10 ps, and we note that this is the same temporal window where the main maximum of the SVHF shows its first plateau. This plateau, $\phi_{\mathrm{MSD}}^{1}∼0.003$ nm${}^{2}$ corresponds to a linear distance of the trapping of the water molecules of ∼0.055 nm, this further proves that in the cage regime at low temperature the particle is trapped inside the cage formed by its neighbours. We also note that the estimation of the cage radius is in agreement with the one done by the position of the main maximum of the SVHFs and the plateau of the SISF.

After the cage relaxes or, in the case of low temperature, the cage-hopping mechanisms set on, the particle can restore the translational motion and correspondently the MSD starts to increase again. Here hydration water molecules move with sub-diffusive motion. In particular, the MSDs show a time-dependence $\propto t^{\delta }$, with δ=0.66.

We then observe here a new second plateau region in some of the MSD curves. In particular this can be seen from T=300 K down to T=230 K. We note that the intervals of temperatures and the time-scale of this second plateau region is the same of the second plateau region of the main maximum of the SVHFs of hydration water, Figure 7. From this second plateau $\phi_{\mathrm{MSD}}^{2}∼$ 0.6 nm${}^{2}$ at T=300 K and ∼0.3 nm${}^{2}$ at T=230 K, we can estimates the length scales at which the particles is transiently trapped again in time: 0.77 and 0.55 nm respectively. This corresponds to the same length scale where we observe the shoulders in the SVHFs of Figure 6. We stress that at variance with the cage-trapping which occurs on the same length scale upon cooling, this second trapping regime is temperature dependent. At high temperature, the hydration water molecules eventually escape from this second trapping, the high-temperature long-time hopping sets on and the MSDs increases again.

## 5. Summary and Conclusions

In this work, we studied in the direct space the dynamical behaviour of the hydration water of lysozyme by analysing the motion of the water molecules within a shell of thickness of 6 Å around the protein. We tracked the dynamics of these molecules through the computation of the self van Hove correlation functions and the mean square displacements at different temperatures ranging from T=300 K upon supercooling down to T=200 K. Correlations times spanned from tens of femtoseconds to tens of nanoseconds.

This analysis revealed the existence of two different caging regimes followed by hopping-dominated structural relaxations. The two mechanisms have different origins. The first process is related to the transient trapping of water molecules by the surrounding neighbours, a feature of the supercooled regime described by the Mode Coupling Theory for the study of glassy dynamics. This process is the same as in bulk water and happens on the same timescale both in bulk and in hydration water.

The water molecules are found to be trapped inside a region with a radius of ∼0.05 nm. This neighbours-caging effect happening at low temperatures, where cages are frozen, prevents in principle the structural relaxation of the liquids. Hopping phenomena then intervene to restore ergodicity. This is a hopping regime characterised by a short length scale (2–3 water shells) and a long-time scale (tens of nanoseconds) and it happens only in the supercooled regime where the structural α relaxation times of hydration water shows strong behaviour, and therefore is related to the fragile to strong transition of the α-relaxation time.

The second caging regime was observed in our calculations at high temperatures, and the cage radius is about 10 times larger than that of the neighbours cage and, differently from the latter, it increases with the temperature (radius of ∼0.77 and 0.55 nm at T=300 and 230 K respectively). The water molecules in the hydration layer can escape this trapping by entering the second hopping regime, which is characterised by a longer length scale (0.8–3 nm) and by the appearance at high temperatures on a shorter time scale (nanoseconds) with respect to the bulk-like hopping phenomena characterising the low temperature region. Given the characteristics of this mechanism we relate it to the protein motions, and in particular to transient trapping of water molecules performed by the protein through hydrogen bonding. The water molecules hydrogen bonded to the protein surface may have inaccessible single particle translational motion but can move with the protein, following its domain rearrangements. This protein-related hopping phenomenon is strictly connected to the protein motion, and in fact we observe it at shorter times above the protein dynamical transitions.

## Abbreviations

The following abbreviations are used in this manuscript:

MCT	Mode Coupling Theory
MD	Molecular Dynamics
MSD	Mean Square Displacement
PDT	Protein Dynamical Transition
SISF	Self Intermediate Scattering Function
SVHF	Self van Hove Function

## References

[1] Rupley J.A., Careri G. (1991). Protein hydration and function. Adv. Protein Chem..

[2] Ball P. (2008). Water: Water—An enduring mystery. Nature.

[3] Franks F. (2000). Water: A Matrix of Life.

[4] Perticaroli S., Comez L., Sassi P., Paolantoni M., Corezzi S., Caponi S., Morresi A., Fioretto D. (2015). Hydration and aggregation of lysozyme by extended frequency range depolarized light scattering. J. Non-Cryst. Solids.

[5] Comez L., Lupi L., Morresi A., Paolantoni M., Sassi P., Fioretto D. (2013). More Is Different: Experimental Results on the Effect of Biomolecules on the Dynamics of Hydration Water. J. Phys. Chem. Lett..

[6] Gallo P., Sciortino F., Tartaglia P., Chen S.H. (1996). Slow Dynamics of Water Molecules in Supercooled States. Phys. Rev. Lett..

[7] Sciortino F., Gallo P., Tartaglia P., Chen S.H. (1996). Supercooled water and the kinetic glass transition. Phys. Rev. E.

[8] Torre R., Bartolini P., Righini R. (2004). Structural relaxation in supercooled water by time-resolved spectroscopy. Nature.

[9] Comez L., Paolantoni M., Sassi P., Corezzi S., Morresi A., Fioretto D. (2016). Molecular properties of aqueous solutions: A focus on the collective dynamics of hydration water. Soft Matter.

[10] Magno A., Gallo P. (2011). Understanding the Mechanisms of Bioprotection: A Comparative Study of Aqueous Solutions of Trehalose and Maltose upon Supercooling. J. Phys. Chem. Lett..

[11] Cerveny S., Swenson J. (2019). Water dynamics in the hydration shells of biological and non-biological polymers. J. Chem. Phys..

[12] Pal S.K., Peon J., Zewail A.H. (2002). Biological water at the protein surface: Dynamical solvation probed directly with femtosecond resolution. Proc. Natl. Acad. Sci. USA.

[13] Zhang L., Wang L., Kao Y.T., Qiu W., Yang Y., Okobiah O., Zhong D. (2007). Mapping hydration dynamics around a protein surface. Proc. Natl. Acad. Sci. USA.

[14] Costard R., Heisler I.A., Elsaesser T. (2014). Structural dynamics of hydrated phospholipid surfaces probed by ultrafast 2D spectroscopy of phosphate vibrations. J. Phys. Chem. Lett..

[15] Siebert T., Guchhait B., Liu Y., Fingerhut B.P., Elsaesser T. (2016). Range, Magnitude, and Ultrafast Dynamics of Electric Fields at the Hydrated DNA Surface. J. Phys. Chem. Lett..

[16] Otting G., Liepinsh E., Wuthrich K. (1991). Protein hydration in aqueous solution. Science.

[17] Bizzarri A.R., Cannistraro S. (2002). Molecular Dynamics of Water at the Protein-Solvent Interface. J. Phys. Chem. B.

[18] Sheu S.Y., Liu Y.C., Zhou J.K., Schlag E.W., Yang D.Y. (2019). Surface Topography Effects of Globular Biomolecules on Hydration Water. J. Phys. Chem. B.

[19] Doster W., Cusack S., Petry W. (1990). Dynamic instability of liquidlike motions in a globular protein observed by inelastic neutron scattering. Phys. Rev. Lett..

[20] Doster W., Cusack S., Petry W. (1989). Dynamical transition of myoglobin revealed by inelastic neutron scattering. Nature.

[21] Combet S., Zanotti J.M. (2012). Further evidence that interfacial water is the main “driving force” of protein dynamics: A neutron scattering study on perdeuterated C-phycocyanin. Phys. Chem. Chem. Phys..

[22] Schirò G., Weik M. (2019). Role of hydration water in the onset of protein structural dynamics. J. Phys. Condens. Matter.

[23] Chen S.H., Liu L., Fratini E., Baglioni P., Faraone A., Mamontov E., Fomina M. (2006). Observation of fragile-to-strong dynamic crossover in protein hydration water. Proc. Natl. Acad. Sci. USA.

[24] Mallamace F., Chen S.H., Broccio M., Corsaro C., Crupi V., Majolino D., Venuti V., Baglioni P., Fratini E., Vannucci C. (2007). Role of the solvent in the dynamical transitions of proteins: The case of the lysozyme-water system. J. Chem. Phys..

[25] Lagi M., Chu X.Q., Kim C., Mallamace F., Baglioni P., Chen S.H. (2008). The low-temperature dynamic crossover phenomenon in protein hydration water: Simulations vs experiments. J. Phys. Chem. B.

[26] Chen S.H., Lagi M., Chu X.Q., Zhang Y., Kim C., Faraone A., Fratini E., Baglioni P. (2010). Dynamics of a globular protein and its hydration water studied by neutron scattering and MD simulations. Spectrosc. Int. J..

[27] Corradini D., Strekalova E.G., Stanley H.E., Gallo P. (2013). Microscopic mechanism of protein cryopreservation in an aqueous solution with trehalose. Sci. Rep..

[28] Camisasca G., De Marzio M., Corradini D., Gallo P. (2016). Two structural relaxations in protein hydration water and their dynamic crossovers. J. Chem. Phys..

[29] Camisasca G., De Marzio M., Rovere M., Gallo P. (2017). Slow Dynamics and Structure of Supercooled Water in Confinement. Entropy.

[30] Camisasca G., Iorio A., De Marzio M., Gallo P. (2018). Structure and slow dynamics of protein hydration water. J. Mol. Liq..

[31] Götze W. (2009). Complex Dynamics of Glass-Forming Liquids.

[32] Starr F.W., Sciortino F., Stanley H.E. (1999). Dynamics of simulated water under pressure. Phys. Rev. E.

[33] Gallo P., Amann-Winkel K., Angell C.A., Anisimov M.A., Caupin F., Chakravarty C., Lascaris E., Loerting T., Panagiotopoulos A.Z., Russo J. (2016). Water: A Tale of Two Liquids. Chem. Rev..

[34] Paschek D., Geiger A. (1999). Simulation Study on the Diffusive Motion in Deeply Supercooled Water. J. Phys. Chem. B.

[35] De Marzio M., Camisasca G., Rovere M., Gallo P. (2017). Microscopic origin of the fragile to strong crossover in supercooled water: The role of activated processes. J. Chem. Phys..

[36] Camisasca G., Galamba N., Wikfeldt K.T., Pettersson L.G.M. (2019). Translational and rotational dynamics of high and low density TIP4P/2005 water. J. Chem. Phys..

[37] Shinohara Y., Dmowski W., Iwashita T., Wu B., Ishikawa D., Baron A.Q.R., Egami T. (2018). Viscosity and real-space molecular motion of water: Observation with inelastic X-ray scattering. Phys. Rev. E.

[38] Sastry S., Debenedetti P.G., Stillinger F.H. (1998). Signatures of distinct dynamical regimes in the energy landscape of a glass-forming liquid. Nature.

[39] Gallo P., Pellarin R., Rovere M. (2003). Slow dynamics of a confined supercooled binary mixture: Direct space analysis. Phys. Rev. E.

[40] Attili A., Gallo P., Rovere M. (2005). Mode coupling behavior of a Lennard-Jones binary mixture: A comparison between bulk and confined phases. J. Chem. Phys..

[41] Roberts C.J., Debenedetti P.G. (1999). Structure and Dynamics in Concentrated, Amorphous Carbohydrate-Water Systems by Molecular Dynamics Simulation. J. Phys. Chem. B.

